# EFFICACY OF SELECTIVE NEUROTOMY FOR FOCAL LOWER LIMB SPASTICITY: A SYSTEMATIC REVIEW

**DOI:** 10.2340/jrm.v56.39947

**Published:** 2024-09-10

**Authors:** Danique J. M. PLOEGMAKERS, Hanneke J. R. VAN DUIJNHOVEN, Liron S. DURAKU, Erkan KURT, Alexander C. H. GEURTS, Tim DE JONG

**Affiliations:** 1 Department of Rehabilitation, Radboud University Medical Center, Nijmegen, the Netherlands; 2 Rehabilitation Centre Klimmendaal, Arnhem, the Netherlands; 3 Department of Plastic, Reconstructive & Hand Surgery, Amsterdam University Medical Center, Amsterdam, the Netherlands; 4 Department of Neurosurgery, Radboud University Medical Center, Nijmegen, the Netherlands; 5 Department of Plastic & Reconstructive Surgery, Radboud University Medical Center, Nijmegen, the Netherlands

**Keywords:** functional neurosurgery, lower extremity, neurotomy, peripheral nerves, spasticity, upper motor neuron syndrome

## Abstract

**Objective:**

Selective neurotomy has been suggested as a permanent treatment for focal spasticity. A systematic literature review was performed to investigate the efficacy of selective neurotomy regarding focal lower limb spasticity.

**Methods:**

A systematic search in PubMed, Medline, Cochrane, and Embase databases was carried out. Studies were included if they reported on the following outcomes: muscle tone, muscle strength, pain, ankle range of motion and/or walking speed, after selective lower limb neurotomy in any type of upper motor neuron syndrome.

**Results:**

A total of 25 non-randomized and/or uncontrolled studies and 1 randomized controlled study were selected. The included studies reported improvements in terms of leg muscle tone, pain, passive range of ankle motion, and walking speed.

**Conclusion:**

The results suggest that selective neurotomy is effective for reducing lower limb spasticity, without any negative effects on walking speed. However, this conclusion is primarily based on uncontrolled case series, whereas conclusions on clinical efficacy should preferably be based on comparison with a reference treatment through (randomized) controlled trials. Future studies should also include quantitative, validated functional assessment tools to further establish the efficacy of selective neurotomy as long-lasting treatment for patients with focal lower limb spasticity.

## LAY ABSTRACT

Selective neurotomy is a neurosurgical intervention whereby a nerve is completely or partially cut to reduce the overactivity of spastic muscles. Selective neurotomy has been suggested as a permanent treatment for focal spasticity of the lower extremities as lower limb spasticity often causes functional problems with standing and walking that hinder a person’s ability to participate in daily activities, resulting in a decreased quality of life. We performed a systematic literature review to assess the efficacy of all types of selective lower limb neurotomy as a treatment for focal spasticity, focusing on walking speed, besides impairment-based measures such as leg muscle tone, leg muscle strength, ankle range of motion, and pain. Our results suggest that selective neurotomy is effective for reducing focal lower limb spasticity, without any negative effects on walking speed, which can be expected when (partially) severing a motor nerve.

Upper motor neuron (UMN) syndromes can lead to various disabling symptoms including spasticity. Lower limb spasticity may cause gait problems, such as knee hyperextension, scissoring of the legs, ankle clonus, toe clawing, and/or pain. These problems can hinder a person’s ability to participate in daily activities, resulting in a decreased quality of life. Spasticity treatment can be categorised into focal or systemic treatments, where focal treatment targets specific nerves or muscles and systemic treatment involves interventions that affect the entire neuromuscular system.

For focal spasticity treatment, both chemodenervation and selective neurotomy can be considered. Intramuscular botulinum toxin administration is the most commonly applied form of focal chemodenervation (1, 2). Its effects are reversible and last about 3 months, which is why this treatment must be regularly repeated. In contrast, selective neurotomy of motor branches innervating spastic muscles has a long-lasting irreversible effect.

The application of peripheral neurotomy was introduced in 1887 by Lorenz (3), and in 1913 Stoffel (4) published on this technique with regard to the upper limb. Despite these early studies, peripheral neurotomy was abandoned in favour of oral medication and chemodenervation due to the adverse effects of the then non-selective neurotomy, such as sensory loss and neuropathic pain. Peripheral neurotomy became more popular again with the advancement of neuroanatomical knowledge and the possibility of intraoperative electrical stimulation, enabling (partial) selective neurotomy of motor nerve branches. It was Brunelli and Brunelli (5) in 1983 who revisited the peripheral nerve surgery procedure for the upper limb and recommended more extensive nerve resection at the primary neurotomy to prevent reinnervation through axonal sprouting from adjacent motor axon terminals.

Neurotomy (sectioning of a nerve trunk) has been proposed for non-functional upper limbs with severe spasticity to facilitate hygiene, nursing, and to improve cosmesis (6). Additionally, selective neurotomy has been proposed for functional upper limbs to decrease the spastic component of a deformity, while retaining some active control of the involved muscles. For the lower limb, the majority of patients pursue functional goals related to their standing and walking capacity, which necessitates taking into account the effect of selective neurotomy on leg muscle strength and functional capacity. In 2011, Bollens et al. (7) performed a systematic review on the efficacy of selective tibial nerve neurotomy in adult patients with spastic equinovarus foot deformity. They described that muscle tone was clinically reduced in all cases, that walking speed improved, and that functional capacity significantly improved. This study suggested that tibial nerve neurotomy is a safe and efficient treatment for these patients. Because this study focused on the spastic foot, they included only studies on selective neurotomy of the tibial nerve. Therefore, we performed an extended systematic literature review containing the most recent studies to assess the efficacy of all types of selective (partial) lower limb neurotomies as a treatment for focal spasticity, focusing on walking speed, besides impairment-based measures such as leg muscle tone, leg muscle strength, ankle range of motion, and pain.

## METHODS

### Research protocol and registration

The protocol for this systematic review was defined in advance and registered in an international database (PROSPERO, registration number CRD42022348103).

### Search strategy and study selection

A biomedical information specialist performed a comprehensive systematic search. The PubMed, Medline, Cochrane, and Embase databases were searched for English papers up to 19 April 2024 for the search string (see Appendix 1). Bibliographic references of identified articles were searched to include additional studies. Articles were first selected based on title and abstract by 2 authors (TdJ and LD). Thereafter, a second screening was conducted based on reading the full paper (TdJ and DP).

### Eligibility criteria

The following inclusion criteria were applied:

- writing in English;
- reporting on results of selective lower limb neurectomy or neurotomy in any type of UMN syndrome;
- including at least 5 subjects;
- clearly describing study protocol;
- reporting one or more of the following outcomes: measures of muscle tone (e.g., [modified] Ashworth Scale [(M)AS]), muscle strength (e.g., Medical Research Council [MRC] scale), pain (e.g., visual analogue scale [VAS], numeric rating scale [NRS], or questionnaire), passive ankle range of motion, walking speed (e.g., 10-meter or 6-Minute Walk Test).

When multiple papers were published on the same study group, the one including the largest study sample or the most recent study was selected.

Review articles, conference letters, or abstracts were excluded.

### Data extraction

Due to a lack of (randomized) controlled trials, we did not apply a formal checklist (e.g., CONSORT) to assess the included studies, nor did we perform any meta-analysis. Instead, 2 authors (DP and TdJ) independently performed risk of bias and GRADE assessment, extracted the available outcomes from the individual studies, and categorized them in tables per type of outcome.

## RESULTS

**Fig. 1:**
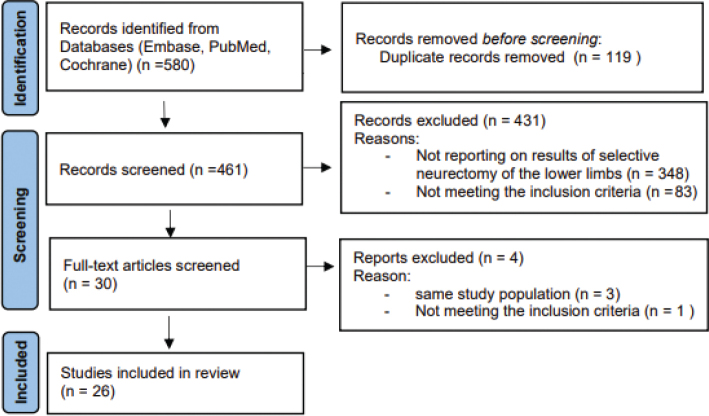
Preferred items for reporting of systematic reviews and meta-analyses (PRISMA) flow diagram.

Of 580 articles identified in the various databases, 119 were removed for being duplicates, 348 were excluded based on content (after reading title and/or abstract), and 84 for not meeting other inclusion criteria (after reading full text). Another 3 articles were excluded because they were based on the same study group. Finally, 26 studies were included in this systematic review (Fig. 1).

A total of 766 participants were included (mean age 42 years, range 4–83 years). The 26 studies together described a total of 10 different types of neurotomy: neurotomy of the tibial nerve (7 studies) (8–14), selective neurotomy of the tibial nerve branches to the soleus muscle (18 studies) (15–32), gastrocnemius muscle (15 studies) (16, 18–26, 28–32), posterior tibial muscle (17 studies) (15–22, 24–32), flexor digitorum longus muscle (11 studies) (11, 16–20, 24, 25, 27, 29, 30), and/or flexor hallucis longus muscle (13 studies) (11, 16–22, 24, 25, 27, 29, 32). Additionally, some studies performed neurotomy of the sciatic nerve (3 studies) (8, 10, 12), selective neurotomy of sciatic branches to the hamstrings (1 study) (11), selective neurotomy of femoral nerve branches to the knee extensors (3 studies) (11, 12, 33), and/or selective neurotomy of obturator nerve branches to the hip adductors (3 studies) (10–12). The design of the 26 studies, the number of included patients, type of intervention, type of outcomes, and length of follow-up are presented in Table I. Of the 26 studies, 16 (9–12, 14–18, 21, 22, 25, 26, 30, 32, 33) reported on previous treatment, none of the patients had a history of neurotomy or phenol treatment, and almost all patients had received at least 1 form of spastic treatment including botulinum toxin, oral antispastic medication, or a baclofen pump. Table II provides the results per type of outcome (leg muscle tone, muscle strength, pain, ankle range of motion, and walking speed). Tables III–VII provide the results in more detail.

**Table I:** Methodological characteristics of the selected studies

Author (year)	Study design	Patients (*n*)	Age (years) Mean [range]	Diagnosis (*n*)	Location of neurotomy (*n*)	Follow-up duration, mean (range)	Outcomes	Grade
Abdennebi (1996)	Retrospective case series	58	24 [5–65]	Cerebral palsy (24), cerebral trauma (4), spinal cord trauma (12), stroke (6), multiple sclerosis (4), encephalitis (3), tumour (2), hydrocephalus (2), echinococcus (1)	Tibialis (66), sciatic (20)	4.2 y	Pain	4
Bollens (2013)	Randomized controlled trial	8	50 [32–70]	Stroke (8)	Soleus (8), TP (4)	6 m	MAS, MRC, PROM, walking speed	2B
Buffenoir (2004)	Prospective pre–post design	55	44 [12–74]	Stroke (34), cerebral trauma (8), spinal cause (7), cerebral palsy (4), other (2)	Soleus (55), TP (39), FDL (36), FHL (28), gastrocnemius (?)	10m (4–22 m)	PROM, walking speed	4
Buffenoir (2008)	Prospective pre–post design	7	41 [19–71]	Stroke (4), head injury (2), postnatal hemiplegia (1)	Superior soleus (7), inferior soleus (5), TP (7), FHL (6), FDL (5)	1 m	PROM, walking speed	4
Buffenoir (2013)	Prospective before– post design	15	47 [22–66]	Stroke (9), head injury (4), amyotrophic lateral sclerosis (1), syringomyelia (1)	Superior soleus (15), inferior soleus (10), GL (3), TP (9), FHL (7), FDL (7)	15 m	MAS, PROM, pain	4
Dauleac (2022)	Prospective before– post design	104	48 [SD 14)	Stroke (70), cerebral trauma (13), encephalic lesion (12), spinal cord injury (7), postoperative brain surgery (2)	Soleus, GM, GL, TP, FHL, FDL	1 y	MAS, MRC Pain	4
Decq (2000)	Prospective before– post design	46	36 [8–79]	Stroke (18), cerebral trauma (15), Little disease (8), mixed disease (5)	Soleus (21), gastrocnemius (9), TP (18), FHL (16), FDL (17)	15m (8–28 m)	MAS, PROM, walking speed	4
Deltombe (2015)	Prospective before– post design	30	45 [20–69]	Stroke (25), cerebral trauma (5)	Soleus (30), GM and GL (16), TP (26), FHL (22)	2 y	AS, MRC, PROM, walking speed	4
Deltombe (2008)	Prospective before– post design	11	51 [38–57]	Stroke (10), cerebral trauma (1)	Soleus (11), TP (10), gastrocnemius (9), FHL (8)	1 y	AS, MRC, PROM, walking speed	4
Feve (1997)	Prospective before– post design	12	36 [6–70]	Stroke (6), head injury (5), spinal injury (1)	Soleus (12), GM and GL (12)	1 m	MRC, PROM, walking speed	4
Fouad (2011)	Retrospective case series	16	47 [18–65]	Stroke (10), cerebral palsy (2), head injury (2), spinal cord injury (2)	Soleus (16), TP (16), gastrocnemius (4), toe flexors (10)	2 y (1–4 y)	MAS, pain	4
Gross (2016)	Prospective before– post design	7	47 [36–54]	Stroke (3), spinal cord injury (4)	Rectus femoris (7)	3 m	MAS, MRC, walking speed	4
Kim (2010)	Retrospective case series	32	Adults 31 [17–51] Children 8 (5–15y)	Stroke (5), cerebral palsy (19), cerebral trauma (7), spinal dysraphism (1)	Tibialis (45)	Adults: 37 m (12–56 m), Children: 43 m (14–96m)	AS, PROM	4
LaMarca (2023)	Prospective before– post design	13	52 [27–67]	Stroke (10), spinal cord injury (1), brain tumour (1), multiple sclerosis (1)	Soleus, GM, GL, TP, FHL, FDL	6m (1–11 m)	Pain Walking speed	4
Lamora (2023)	Retrospective case series	46	46 [20–79]	Stroke (30), cerebral trauma (6), stroke+cerebral trauma (3), hypoxia (1), other (6)	Soleus (47), gastrocnemius (13), TP (34), FHL	2 m/22 m** (13–184 d) / (204–555 d)	MAS, MRC, PROM	4
Le Bocq (2016)	Prospective before– post design	23	57 [48–63]	Stroke (23)	Soleus (23), gastrocnemius (23), TP (10)	5 m	MAS, MRC, PROM, walking speed	4
Liu (2022)	Prospective before– post design	14	43 [24–65]	Spinal cord injury (14)	Sciatic (4), tibialis (26), obturator (26)	1.5 m	MAS, walking speed	4
Mahan (2021)	Retrospective case series	38*	49 [22–74]	Stroke (22), cerebral trauma (6), anoxic injury (2), cerebral palsy (3), spinal cord injury (3), brainstem tumour (1), progressive multifocal leukoencephalopathy (1)	Plantar flexor (15), toe flexor (1), hip adductors (2), knee extensors (2), hamstrings (2), hip flexors (2)	16 m (1–59 m)	MAS	4
Oda (2022)	Retrospective case series	26	60 [16–83]	Stroke (24), cerebral trauma (2)	Soleus (26), TP (25), FHL (3), FDL (11)	6 m	MAS, MRC, walking speed, pain	4
Palacio (2010)	Retrospective case series	25	55 [36–81]	Stroke (17), head injury (7), thrombophlebitis (1)	Soleus (23), gastrocnemius (24), TP (1)	11 y 3 m (4–19 y)	MAS	4
Roujeau (2003)	Retrospective case series	6	28 y	Hereditary spastic paraplegia (1), prematurity (1), Arnold-Chiari malformation (1), stroke (1), head injury (2)	Soleus (7), gastrocnemius (7), TP (1), FHL (2), FDL (2)	29 m (10–48 m)	PROM	4
Rousseaux (2009)	Prospective before– post design	51	51	Stroke (51)	Soleus (51), gastrocnemius (50), TP (27), FDL (10)	2 y	MAS, MRC, PROM, walking speed	4
Salem (2018)	Prospective before– post design	20	31	Stroke (4), cerebral palsy (3), cerebral trauma (5), spinal cord injury (4), spinal tumour (2), hereditary spastic paraplegia (2)	Sciatic (15), tibialis (28), obturator (15), femoral (2)	1 y	MAS, MRC, ROM	4
Sindou (1985)	Retrospective case series	39	35 [6–68]	Brain lesion (25), spinal cord lesion (14)	Tibialis	4 y (1–9 y)	Pain	4
Sindou (1988)	Retrospective case series	53	36 [6–68]	Stroke (19), brain injury (14), brain tumour (1), meningo-encephalitis (1), cerebral palsy (6), spinal cord lesion (12)	Tibialis (62)	3 y (15 m–10 y)	AS, PROM, pain	4
Sitthinamsuwan (2013)	Retrospective case series	11***	18 [4–48]	Cerebral trauma (5), cerebral palsy (4), encephalitis (1), arteriovenous malformation (1)	Soleus (14), gastrocnemius (14), TP (2)	40 m (24–52 m)	MAS, PROM	4

N: number, d: days, m: months, y: years, SD: standard deviation, M: male, MRC: Medical Research Council. (M)AS: (modified) Ashworth scale. PROM: passive range of motion. GM: gastrocnemius medial, GL: gastrocnemius lateral, TP: tibialis posterior, FHL: flexor hallucis longus, FDL: flexor digitorum longus.

* Lower and upper extremity.

** 46 patients <6 m follow-up, 24 patients >6 m follow up.

*** Total number of patients is 15, we excluded the 4 patients who underwent combined selective tibial neurotomy and tendo Achilles lengthening.

**Table II:** Outcomes of the included studies

Author	Muscle tone (M)AS	Muscle strength (MRC)	PROM ankle	Walking speed	Pain
Abdennebi (1996)					↑
Bollens (2013)	↑*	Ø	Ø	Ø	
Buffenoir (2004)			↑*	↑*	
Buffenoir (2008)			↑*	↑*	
Buffenoir (2013)	↑*		↑*		↑
Dauleac (2022)	↑*	Ø			↑
Decq (2000)	↑		Ø	Ø	
Deltombe (2015)	↑*	Ø / ↑* §	Ø	↑*	
Deltombe (2008)	↑*	↓*	↑*	Ø	
Feve (1997)		↓	Ø	Ø	
Fouad (2011)	↑				↑
Gross (2016)	↑	Ø		↑*	
Kim (2010)	↑		↑		
LaMarca (2023)				↑*i	↑
Lamora (2023)	↑*	Ø	Ø		
Le Bocq (2016)	↑*	↓/ ↑* ‡	↑*	Ø / ↑* †	
Liu (2022)	↑*			Ø	
Mahan (2021)	↑*				
Oda (2022)	↑*	Ø / ↑* §		↑*	↑*
Palacio (2010)	↑				
Roujeau (2003)			Ø		
Rousseaux (2009)	↑*	↓* / ↑* ‡	↑*	↑*	
Salem (2018)	↑*	↑*	↑*¤		
Sindou (1985)					↑
Sindou (1988)	↑		↑		↑
Sitthinamsuwan (2013)	↑*		↑*		

↑ *Statistically significant improvement.

↓ *Statistically significant deterioration.

↑ Improvement for more than 50% of patients, in absence of statistical analysis.

↓ Deterioration for more than 50% of patients, in absence of statistical analysis.

Ø No statistical difference, or modification for less than 50% of patients in absence of statistical analysis.

‡ MRC ankle plantar flexors decrease, MRC ankle dorsal flexors increase.

§ MRC ankle plantar flexors unchanged, MRC ankle dorsal flexors increase.

↑ *iSignificant increase in self-selected walking speed, increase in maximum walking speed but not significant.

† No significant change in comfortable walking speed, but significant increase in maximum walking speed.

¤ ROM was performed only for muscle groups involved that underwent neurosurgical intervention, not especially ankle ROM. Unknown whether active or passive ROM.

**Table III:** Effects of peripheral neurotomy on leg muscle tone in the selected studies

Author	Location of neurotomy	Muscle	(M)AS unchanged*	(M)AS decreased*
Bollens 2013	8 soleus, 4 TP, 4FHL	Triceps surae Soleus		Significant decrease triceps surae (MAS from 3 to 2, *p* = 0.010) and soleus (MAS from 3 to 0, *p* < 0.001)
Buffenoir 2013	15 superior soleus, 10 inferior soleus, 3 GL, 9 TP, 7 FHL, 7 FDL	Triceps surae Soleus		Significant decrease triceps surae (MAS from 2 to 0, *p* < 0.001) and soleus (MAS from 2 to 0, *p* < 0.001)
Dauleac 2022	Soleus, GM, GL, TP, FHL, FDL	Triceps surae Tibialis posterior FHL, FDL		Significant decrease triceps surae, tibialis posterior, FHL and FDL (all MAS from 3 to 0, *p* < 0.0001)
Decq 2000	21 soleus, 9 gastrocnemius, 18 TP, 16 FHL, 17 FDL	Triceps surae Soleus	2 patients continued to have clonus of the gastrocs	44 patients (95%) MAS decreased from 2 to 0
Deltombe 2015	30 soleus, 16 GM and GL, 26 TP, 22 FHL	Triceps surae Tibialis posterior Quadriceps Hamstrings	No significant change quadriceps (AS 2) hamstrings (AS 2 to 1)	Significant decrease in triceps surae (AS 3 to 1, *p* < 0.001) and tibialis posterior (AS 1 to 0, *p* < 0.001)
Deltombe 2008	11 soleus, 10 TP, 9 gastrocnemius, 8 FHL	Triceps surae Quadriceps Hamstrings	No significant change quadriceps (AS 2) hamstrings (AS 1)	Significant decrease in triceps surae (AS 3 to 0, *p* = 0.003)
Fouad 2011	16 soleus, 16 TP, 4 gastrocnemius, 10 toe flexors		1 patient same MAS	Reduction of MAS in 8 patients ≥ –3, in 5 patients –2, in 2 patients –1
Gross 2016	7 rectus femoris	Quadriceps	1 patient same MAS	Reduction of MAS in 6 patients with median MAS from 2 to 1 (*p* = 0.421)
Kim 2010	45 tibialis	Triceps surae		Mean AS in adults from 3.6 to 1.6. In children from 3.7 to 1.4
Lamora 2023	47 soleus, 13 gastrocnemius, 34 TP, FHL	Triceps surae Soleus		Significant decrease triceps surae (MAS from 3 to 2, *p* < 0.001) and soleus (MAS from 2 to 0, *p* < 0.001)
Le Bocq 2016	23 soleus, 23 gastrocnemius, 10 TP	Triceps surae Tibialis posterior Quadriceps Hamstrings	No significant change quadriceps (MAS 2) hamstrings (MAS 1)	Significant decrease in triceps surae (MAS 4 to 0. *p* < 0.0001) and tibialis posterior (MAS 2 to 1, *p* < 0.0001)
Lui 2022	4 sciatic, 26 tibialis, 26 obturator	Hip adductor Triceps surae Hamstrings		Significant decrease in all 3 muscles MAS 4 to 1 (*p* < 0.01)
Mahan 2021	15 plantar flexor, 1 toe flexor, 2 hip adductors, 2 knee extensors, 2 hamstrings, 2 hip flexors	Muscle group that underwent surgery		Significant decrease in mean MAS 3 to 1 (*p* < 0.001)
Oda 2022	26 soleus, 25 TP, 3 FHL, 11 FDL	Ankle dorsal flexors Ankle plantar flexors		Significant decrease in dorsal flexors (MAS 0.4 to 0.1, *p* < 0.001) and plantar flexors (MAS 2.2 to 1.2, *p* < 0.001)
Palacio 2010	23 soleus, 24 gastrocnemius, 1 TP	Triceps surae Soleus Tibialis posterior	1 case MAS 3	22 cases MAS 0, 2 cases MAS 1
Rousseaux 2009	51 soleus, 50 gastrocnemius, 27 TP, 10 FDL	Ankle plantar flexors		Significant decrease in MAS 3 to 1 (*p* = 0.0001)
Sindou 1988	62 tibialis	Triceps surae		Mean AS from 3.8 to 1.5
Salem 2018	15 sciatic, 28 tibialis, 15 obturator, 2 femoral	Muscle group that underwent surgery		Significant decrease in MAS 3.6 to 2.3 (*p* < 0.001)
Sitthinamsuwan 2013	14 soleus, 14 gastrocnemius, 2 TP			Mean MAS (in patients without tendo Achilles lengthening) from 2.6 to 0.2

GM: gastrocnemius medial GL: gastrocnemius lateral; TP: tibialis posterior; FHL: flexor hallucis longus; FDL: flexor digitorum longus. (M)AS: modified Ashworth scale.

* At end of follow-up.

**Table IV:** Effects of peripheral neurotomy on leg muscle strength in the selected studies

Author	Location of neurotomy	Muscle	MRC unchanged*	MRC decreased*	MRC increased*
Bollens 2013	8 soleus, 4 TP, 4FHL	Triceps surae Tibialis anterior	Triceps and TA no significant change in strength after 6 m		
Dauleac 2022	Soleus, GM, GL, TP, FHL, FDL	Triceps surae Tibialis posterior FHL, FDL	Triceps, TP, FHL and FDL no significant change in strength		
Deltombe 2015	30 soleus, 16 GM and GL, 26 TP, 22 FHL	Triceps surae Tibialis anterior	Triceps no significant change in strength after 2 y		Tibialis anterior (antagonist): significant increase after 2 y
Deltombe 2008	11 soleus, 10 TP, 9 gastrocnemius, 8 FHL	Triceps surae		Significant decrease (–1 MRC)	
Feve 1997	12 soleus, 12 GM and GL	Triceps surae	1 patient (untestable)	11 patients (–2 or 3 MRC)	
Gross 2016	7 rectus femoris	Hip flexors Knee extensors	Hip flexors and knee extensors no significant change		
Lamora 2023	47 soleus, 13 gastrocnemius, 34 TP, FHL	Triceps surae Ankle dorsal flexors	Triceps surae and ankle dorsal flexors no significant change		
Le Bocq 2016	23 soleus, 23 gastrocnemius, 10 TP	Triceps surae Tibialis anterior		Triceps non-significant decrease (–1MRC)	Tibialis anterior (antagonist): significant increase (+1MRC) (*p* = 0.007)
Oda 2022	26 soleus, 25 TP, 3 FHL, 11 FDL	Ankle dorsal flexors Ankle plantar flexors	Ankle plantar flexors no significant change		Ankle dorsal flexors (antagonist): significant increase (+0.2MRC) (*p* = 0.029)
Rousseaux 2009	51 soleus, 50 gastrocnemius, 27 TP, 10 FDL	Ankle plantar flexors Ankle dorsal flexors		Ankle plantar flexors significant decrease (–0.3MRC) (*p* < 0.01)	Ankle dorsiflexors (antagonist) significant increase (+0.5MRC)
Salem 2018	15 sciatic, 28 tibialis, 15 obturator, 2 femoral	Muscle group that underwent surgery			Significant increase (+0.3MRC) (*p* = 0.041) 5 (25%) showed improvement on MRC

MRC: Medical Research Council; GM: gastrocnemius medial; GL: gastrocnemius lateral; TP: tibialis posterior; FHL: flexor hallucis longus; FDL: flexor digitorum longus; y: years.

* At end of follow-up.

**Table V:** Effects of peripheral neurotomy on ankle range of motion in the selected studies

Author	Location of neurotomy	PROM ankle unchanged*	PROM ankle increased*
Bollens 2013	8 soleus, 4 TP, 4FHL	No significant change in PROM after 2 y	
Buffenoir 2004	55 soleus, 39 TP, 36 FDL, 28 FHL, ? gastrocnemius		PROM significantly increased. With flexed knee +5° (*p* = 0.0066). With extended knee +7° (*p* = 0.0001).
Buffenoir 2008	7 superior soleus, 5 inferior soleus, 7 TP, 6 FHL, 5 FDL		PROM significantly increased. With flexed knee 7.1° to 12.1° (*p* < 0.05). With extended knee from 10° to 15.7° (*p* < 0.05)
Buffenoir 2013	15 superior soleus, 10 inferior soleus, 3 GL, 9 TP, 7 FHL, 7 FDL		PROM significantly increased. With extended knee +9° (*p* < 0.001).
Decq 2000	21 soleus, 9 gastrocnemius, 18 TP, 16 FHL, 17 FDL	PROM remained unchanged after surgery	The ankle angle at the end of the second rocker increased statistically significant, ranging from an average of –3.82° to 4.24° (*p*=0.0098).
Deltombe 2015	30 soleus, 16 GM and GL, 26 TP, 22 FHL	No significant increase in PROM after 2 y with flexed and extended knee	
Deltombe 2008	11 soleus, 10 TP, 9 gastrocnemius, 8 FHL		PROM with flexed knee increased significantly (+ 5°, *p* = 0.015)
Feve 1997	12 soleus, 12 GM and GL	PROM remained unchanged after surgery	
Kim 2010	45 tibialis		Mean PROM ankle improved (adults 11.2° pre- and 17.6° postop. Children 11.8° pre- and 22.5° postop)
Lamora 2023	47 soleus, 13 gastrocnemius, 34 TP, FHL	No significant increase in PROM after >6 m with flexed and extended knee	
Le Bocq 2016	23 soleus, 23 gastrocnemius, 10 TP		PROM significantly increased. With flexed knee +10° (*p* < 0.0001). With extended knee +15° (*p* < 0.001) although limitations persisted in 7 cases
Roujeau 2003	7 soleus, 7 gastrocnemius, 1 TP, 2 FHL, 2 FDL	PROM remained unchanged in 5 cases	PROM with extended knee improved in 2 cases
Rousseaux 2009	51 soleus, 50 gastrocnemius, 27 TP, 10 FDL		PROM with extended knee increased significantly (+ 10°, *p* = 0.0001)
Salem 2018	15 sciatic, 28 tibialis, 15 obturator, 2 femoral		Statistically significant improvement at 6 m and 1 y postoperative (*p*<0.001)**
Sindou 1988	62 tibialis		Postoperative return to normal passive dorsiflexion (15°) in 41 cases (i.e., in 77.35% of cases).
Sitthinamsuwan 2013	14 soleus, 14 gastrocnemius, 2 TP		Mean PROM (in patients without tendo Achilles lengthening) increased from 44.3° pre- to 66.4° postop

PROM: passive range of motion; GM: gastrocnemius medial GL: gastrocnemius lateral; TP: tibialis posterior; FHL: flexor hallucis longus; FDL: flexor digitorum longus; y: years; m: months.

* At end of follow-up.

** ROM was performed only for muscle groups involved that underwent neurosurgical intervention; not especially ankle ROM. Unknown whether active or passive ROM.

**Table VI:** Effects of peripheral neurotomy on pain in the selected studies

Author	Location of neurotomy	Patients with preoperative pain	Pain relief	Pain unchanged
Abdennebi 1996	20 sciatic, 66 tibialis	38	29 patients (76%) free of pain 5 patients (13%) mild residual pain	2 patients (5%) severely pain 2 patients (5%) moderately pain
Buffenoir 2013	15 superior soleus, 10 inferior soleus, 3 GL, 9 TP, 7 FHL, 7 FDL	3	3 patients VAS score decreased from 5/10 to 1.3/10	0
Dauleac 2022	Soleus, GM, GL, TP, FHL, FDL	17	13 patients with a goal related to pain corresponded to expected level (2 patients), better outcome (4 patients), or much better outcome (7 patients) than expected according to GAS	4 patients with a goal related to pain had no relief (3 patients) or did not correspond to expected level (1 patient) according to GAS
Fouad 2011	16 soleus, 16 TP, 4 gastrocnemius, 10 toe flexors	4	4 (100%) improved on VAS	
LaMarca 2023	Soleus, GM, GL, TP, FHL, FDL	3	3 patients with an expectation to reduce pain, scored 92.7 on average on a scale from 0–100	
Oda 2022	26 soleus, 25 TP, 3 FHL, 11 FDL	10	Mean NRS decreased significantly from 6.4 to 2.7 (*p* = 0.009) 8 patients showed improvement in pain symptoms	2 patients pain intensity remained the same
Sindou 1985	tibialis	43	40 patients	3 patients
Sindou 1988	62 tibialis	35	33 complete relief of pain 2 significant relief of pain	

TP: tibialis posterior; GL: gastrocnemius lateralis; FHL: flexor hallucis longus; FDL: flexor digitorum longus. GAS: Goal Attainment Scaling. VAS: visual analogue scale; NRS: numeric rating scale.

**Table VII:** Effects of peripheral neurotomy on walking speed in the selected studies

Author	Location of neurotomy	Measurement	Walking speed unchanged	Walking speed increased
Bollens 2013	8 soleus, 4 TP, 4 FHL	10MWT	No influence on spontaneous walking speed	
Buffenoir 2004	55 soleus, 39 TP, 36 FDL, 28 FHL, ? gastrocnemius	10MWT		Walking speed increased significantly at normal speed with wearing shoes or with bare feet (*p* < 0.05)
Buffenoir 2008	7 superior soleus, 5 inferior soleus, 7 TP, 6 FHL, 5 FDL	10MWT		Walking speed increased significantly at normal speed and rapid speed with wearing shoes and with bare feet (*p* < 0.05)
Decq 2000	21 soleus, 9 gastrocnemius, 18 TP, 16 FHL, 17 FDL	Gait analysis by markers	Not significantly modified for spontaneous walking speed (*p* = 0.41)	
Deltombe 2015	30 soleus, 16 GM and GL, 26 TP, 22 FHL	10MWT		Walking speed increased after 2 months (*P* = 0.004) and 2 years (*p* = <0.001)
Deltombe 2008	11 soleus, 10 TP, 9 gastrocnemius, 8 FHL	6MWT	No significant effect on walking speed	
Feve 1997	12 soleus, 12 GM and GL	Video gait analysis	No significant changes in walking speed	
Gross 2016	7 rectus femoris	Video gait analysis		Spontaneous walking speed increased significantly (*p* = 0.02)
LaMarca 2023	Soleus, GM, GL, TP, FHL, FDL	10MWT	No significant change in fast walking speed (*p*=0.11)	Self-selected walking speed increased significantly (*p* = 0.03)
Le Bocq 2016	23 soleus, 23 gastrocnemius, 10 TP	GAITrite mat	No significant change in comfortable walking speed (*p* = 0.245)	Maximum walking speed slightly increased (*p* = 0.008)
Liu 2022	4 sciatic, 26 tibialis, 26 obturator	Gait analysis	No significant increase in walking speed (*p*=0.932)	
Oda 2022	26 soleus, 25 TP, 3 FHL, 11 FDL	10MWT		Walking speed increased significantly (*p* = 0.037)
Rousseaux 2009	51 soleus, 50 gastrocnemius, 27 TP, 10 FDL	10MWT		Improvement in comfortable speed with usual aid (*p* = 0.01) and at rapid speed with aid (*p* = 0.013) 2 years after neurotomy

TP: tibialis posterior; GL: gastrocnemius lateralis; GM: gastrocnemius medialis; FHL: flexor hallucis longus; FDL: flexor digitorum longus; 10MWT: 10 metre walk test; 6MWT: 6 minute walk test.

### Muscle tone

Nineteen studies (9–12, 14–16, 19–22, 24, 26–28, 30–33) (576 participants) examined the change in leg muscle tone by using (M)AS scores (see Table III). In all studies the mean (M)AS decreased in the muscles that underwent surgery to a postoperative score ≤2. Thirteen studies (10–12, 15, 16, 19, 21, 22, 26, 27, 30–32) mentioned that this reduction was statistically significant. Four studies (20, 24, 28, 33) (total 94 participants) mentioned the outcomes per participant, of which 89 participants (95%) showed a reduction of muscle tone in the muscles treated.

### Muscle strength

In 11 studies (12, 15, 19, 21–23, 26, 27, 30, 32, 33) (316 patients) muscle strength was reported (see Table IV). In 8 studies (15, 19, 21, 23, 26, 27, 32, 33) no significant change in muscle strength was observed in the muscles that had undergone surgery, whereas 2 studies reported a statistically significant loss of strength. Deltombe et al. (22) reported that the strength of the triceps surae decreased from MRC score 3.3 preoperatively to 2.3 1 year after selective neurotomy of the tibial nerve. Likewise, in the study of Rousseaux et al. (30), ankle plantar flexor strength decreased from an MRC score of 2.75 to 2.45, 2 years after selective neurotomy of the tibial nerve. Five (12, 21, 26, 27, 30) studies reported a significant increase in muscle strength, but this increase concerned the antagonists to the muscles affected by the neurotomy (i.e., the ankle dorsiflexors) in 4 of the 5 studies. In the remaining study (12) where an increase in muscle strength was reported, this increase was observed in 25% of the patients in the muscle group that underwent surgery, with a significant improvement of +0.3 on the MRC scale.

### Ankle range of motion

Fifteen studies (9, 14–18, 20–23, 26, 29–32) (385 participants) measured passive range of ankle motion (see Table V). All studies concerned selective neurotomy of the tibial nerve. In 9 studies (9, 14, 16–18, 22, 26, 30, 31) (258 participants), ankle range of motion improved by 5 to 22 degrees. Six studies (15, 20, 21, 23, 29, 32) (127 participants) did not show a change in passive range of ankle motion. One other study (12) mentioned a significant positive effect of lower limb neurotomy on joint range of motion, but this improvement did not specifically concern the ankle joint, nor was it clear whether passive or active range of motion was measured.

### Pain

Eight studies (8, 13, 14, 16, 19, 24, 25, 27) (324 participants) reported on pain (see Table VI). All studies concerned selective neurotomy of the tibial nerve, which was combined with the sciatic nerve in 1 study (8). Among all participants, 153 (47%) reported pain preoperatively. Each study mentioned total or partial pain relief after surgery in the majority of the participants. In only 13 patients was there no pain relief. In the study by Oda et al. (27) mean NRS decreased significantly from 6.4 to 2.7, and in the study by Buffenoir et al. (16) VAS pain decreased from 5 preoperatively to 1.3 postoperatively. Fouad also used a VAS score and found that pain improved in all patients with preoperative pain (25%), without mentioning the exact outcomes (24). Dauleac et al. (19) measured the achievement of individual goals using Goal Attainment Scaling (GAS). In this study, 17 patients with a goal related to pain corresponded to expected level (2 patients), better outcome (4 patients), or much better outcome (7 patients) than expected according to the GAS. Three patients had no relief of pain and 1 patient did not correspond to the expected level. LaMarca et al. (25) did a postoperative survey to investigate perceived improvements. Three patients reported perceived improvements of pain with an average score of 92.7 quantified on a 100-point scale. Other studies (8, 13, 14) did not mention how they assessed pain.

### Walking speed

Thirteen studies (10, 15, 17, 18, 20–23, 25–27, 30, 33) (305 participants) measured walking speed using the 10-Meter Walk Test (10MWT), 6-Minute Walk Test (6MWT), or some form of instrumented gait analysis (see Table VII). In 12 studies this concerned selective neurotomy of the tibial nerve, of which 1 study also performed selective neurotomy of the sciatic nerve (10). In the study by Gross et al. it concerned selective neurotomy of the nerve to the rectus femoris (33). Seven studies (189 participants) reported a statistically significant increase in comfortable walking speed. Deltombe et al. and Gross et al. found that comfortable walking speed increased respectively from 0. m/s preoperatively to 0.7 m/s and from 0.65 m/s preoperatively to 0.69 m/s postoperatively (21, 33). Rousseaux et al. (30) reported a similar increase in comfortable speed with a walking aid (from 0.52 m/s to 0.56 m/s) and during fast walking (from 0.64 m/s to 0.72 m/s) 2 years post-surgery. Buffenoir et al. (18) reported an increase in comfortable walking speed, measured by the time taken to walk 10 metres, from 55 to 35 s with bare feet and from 41 to 30 s while wearing shoes. Buffenoir et al. (17) reported similar results, namely the walking time at normal speed with bare feet improved from 46 to 30 s and at rapid speed from 37 to 23 s. Oda et al. (27) also used the 10MWT, with mean scores of 32 seconds preoperatively and 31 seconds postoperatively. One other study mentioned an increase in maximum walking speed (from 0.44 m/s preoperatively to 0.6 1m/s postoperatively), but no significant change in comfortable walking speed (26), while LaMarca et al. (25) reported a significant change in comfortable walking speed (increase from 0.58 m/s preoperatively to 0.70 m/s postoperatively) but not in maximum walking speed. None of the studies reported a decrease in walking speed.

## DISCUSSION

Selective neurotomy is not yet widely known and used as a treatment option for focal lower limb spasticity in people with UMN syndrome, perhaps due to fear of causing deterioration of standing and/or walking capacity after the procedure. In this review we focused on the efficacy of all types of selective neurotomies for focal lower limb spasticity and our results suggest that selective neurotomy is an effective treatment for reducing focal lower limb spasticity without a negative effect on walking speed. In a previous systematic review Bollens et al. showed similar results for neurotomies of the tibial nerve for the spastic foot (7).

All studies (9–12, 14–16, 19, 21, 22, 26, 27, 30) that included the (M)AS as an outcome showed a tone reduction of the muscles treated, while studies (20, 24, 28, 33) that reported the change in (M)AS per participant found decreased muscle tone in 95% of treated patients. In the remaining participants who showed no reduction of muscle tone, this lack of effect mainly concerned the soleus, for which neurotomy had been partially performed or not at all. As 14 of the 19 studies used a follow-up period of at least 1 year, the observed muscle tone improvements appear to be long-lasting effects (9, 11, 12, 14, 16, 19–22, 24, 28, 30–32). Because loss of muscle strength might be an adverse effect of neurotomy, several studies investigated parallel effects of selective neurotomy on muscle strength. Two studies (22, 30) reported a significant decrease in strength of the muscles that underwent surgery. It has been shown that muscle strength decreases immediately after the neurotomy due to the alpha fibres sectioning but increases in the following year due to collateral reinnervation (22). The extent of muscle strength loss differed from a significant reduction of –0.3 (30) to –1 (22) points on the MRC scale. Interestingly, 5 studies (12, 21, 26, 27, 30) reported a significant increase in muscle strength following selective neurotomy (MRC scale + 0.2 to 1.0 points), which in 3 (21, 26, 27, 30) of the 4 studies was observed for the antagonistic muscles (i.e., the ankle dorsiflexors). Apparently, selective neurotomy of the tibial nerve reduces spastic co-contraction of the ankle plantar flexors to such a degree that voluntary contraction of the ankle dorsiflexors will be less obstructed (32).

Our results also support the notion that tibial nerve neurotomy may improve passive range of ankle motion, probably due to reduced muscle resistance upon ankle plantar flexor stretch. Improvements ranging from 5 to 22 degrees were reported in 9 out of 15 studies (9, 14, 16–18, 22, 26, 30, 31). Possibly, improved jont mobility also has a relationship with pain relief as a reduction of plantar (fore)foot pressure. However, it must be acknowledged that the method of assessment in the 5 studies that reported on pain varied substantially or this was not mentioned, which warrants cautious interpretation. Moreover, it is important to keep in mind that there are several factors other than improved passive range of ankle motion or calf muscle tone that may influence pain in people with UMN syndrome. This highlights the need for further prospective research in this area, using well-established and validated pain assessments.

Despite some reduction of muscle strength in the treated muscles, none of the studies reported a decrease in walking speed, while 7 out of 13 studies reported improved walking speed after selective neurotomy (17, 18, 21, 25, 27, 30, 33). In 12 studies (10, 15, 17, 18, 20–23, 25–27, 30) it concerned neurotomy of the tibial nerve and in 1 study (10) also the sciatic nerve. A possible mechanism explaining the preserved or even improved walking speed in all studies – despite the loss of calf muscle strength in some studies – may be the often observed improvements in passive range of ankle motion and/or pain reduction, which allows a more stable and prolonged single-support stance phase on the affected limb during walking (34). Moreover, it is conceivable that reduction of calf muscle tone in combination with improved strength of the ankle dorsiflexors further contributes to a more natural progression of the centre of body mass – and thus to energy conservation – while walking.

To date only 1 small single-blinded RCT has been performed comparing botulinum toxin with selective neurotomy (15). It found that selective tibial neurotomy induced a higher reduction in ankle stiffness compared with botulinum toxin. However, both treatments induced a comparable improvement in ankle kinematics during gait and neither resulted in muscle weakening. Furthermore, both treatment groups showed no significant change in activity, participation, and quality of life.

In 2010, Foley et al. (35) concluded in their meta-analysis that the use of botulinum toxin-A for lower limb post-stroke spastic equinovarus deformity was associated with a small but statistically significant increase in walking speed. In 2008, Rousseaux et al. (36) had already reported that tibial nerve neurotomy is even more effective than botulinum toxin injections for improving gait speed and gait independence (i.e., functional ambulation categories). From a clinical perspective, a selective diagnostic nerve block of the motor nerve branches innervating the targeted spastic muscles is recommendable (21), because this allows detailed professional assessment of the respective contribution of individual muscles to the observed lower limb impairments and loss of functional capacity. Moreover, selective diagnostic nerve blocks provide patients with the opportunity to experience what could be achieved through a neurotomy procedure, which enables shared decision-making. When unsatisfactory or limited improvement is expected based on this work-up neurotomy can be replaced by or combined with orthopaedic interventions such as arthrodesis, tendon-lengthening procedures, and/or tendon transfers (37, 38).

### Limitations

It is important to note that the level of evidence supporting the efficacy of selective lower limb neurotomy is currently limited. Due to methodological limitations, 25 of 26 studies that were included in this review scored “low quality” on methodological assessment, which implies a high risk of bias. The methodological characteristics of the included studies (Table I) highlight 3 major limitations. First, (randomized) controlled trials were rare. It is known that controlled studies are notoriously difficult to perform in people with UMN syndrome who are seeking a more definitive solution for long-lasting complaints and disability due to spasticity. Nevertheless, trials comparing selective neurotomy with a reference treatment are required in order to reach a higher level of scientific evidence, which may imply the need for multi-centre collaboration to obtain sufficient numbers of patients undergoing the same procedure. In addition, such trials should ensure blinding of outcome assessors to prevent measurement bias. Second, the applied outcome measures varied substantially, which prohibits comparisons of outcomes between studies and performing meta-analyses. For future studies, it is important that standardized, widely accepted outcome tools are used with good measurements properties to detect clinically relevant changes in all domains of the International Classification of Functioning, Disability and Health (ICF), including goal attainment regarding daily activities and social participation (39). Instrumented gait analysis could be added to clinical gait measures to obtain a better understanding of the underlying mechanisms leading to functional benefits. Third, the methods of (partial or complete) neurotomy were not always mentioned.

Another limitation of the included studies is that several papers did not describe whether complications or side effects, other than loss of muscle strength, had occurred. Although 15 of the 26 included studies (8, 9, 11–16, 18, 19, 24–26, 28, 30) mentioned 1 or more complications after the surgical intervention, these outcomes were often not well described. The observed complications varied from wound infection, transient oedema, and delayed wound healing to wound dehiscence, complex regional pain syndrome, and sensibility problems (e.g., hyperesthesia or hypesthesia). In addition, recurrence of spasticity and revision surgery was only sometimes mentioned (11, 24). Hence, future studies should provide more detailed reports of the incidence and severity of postoperative complications after selective lower limb neurotomy to optimally inform patients about the safety of the procedure.

### Conclusion

This study provides an overview of the available literature on the efficacy of selective (partial) neurotomy as a treatment for patients with focal lower limb spasticity. The findings of this systematic review suggest that selective neurotomy may be an effective treatment option for lower limb spasticity. The included studies reported positive outcomes regarding leg muscle tone, walking speed, pain, and often also passive range of ankle motion.
